# Clinical relevance of vitamin B12 level and vitamin B12 metabolic gene variation in pulmonary tuberculosis

**DOI:** 10.3389/fimmu.2022.947897

**Published:** 2022-10-06

**Authors:** Tian-Ping Zhang, Rui Li, Li-Jun Wang, Fei Tang, Hong-Miao Li

**Affiliations:** ^1^ Department of Rheumatology and Immunology, The First Affiliated Hospital of USTC, Division of Life Sciences and Medicine, University of Science and Technology of China, Hefei, China; ^2^ Department of Nosocomial Infection Management, The First Affiliated Hospital of Anhui Medical University, Hefei, China; ^3^ Department of Infectious Diseases, The First Affiliated Hospital of Anhui Medical University, Hefei, China; ^4^ Department of Interventional Pulmonology and Endoscopic Diagnosis and Treatment Center, Anhui Chest Hospital, Hefei, China; ^5^ Department of Epidemiology and Biostatistics, School of Public Health, Anhui Medical University, Hefei, China

**Keywords:** pulmonary tuberculosis, infectious disease, Mycobacterium tuberculosis, vitamin B12, single nucleotide polymorphisms

## Abstract

The aim of this study was to assess the association of vitamin B12 level and single nucleotide polymorphisms (SNPs) in vitamin B12 metabolic genes with pulmonary tuberculosis (PTB) in Chinese Han population. The plasma vitamin B12 expression level was detected using ELISA. Ten SNPs in six key genes (*TCN1*, *TCN2*, *CUBN*, *MMACHC*, *FUT6*, and *MUT*) of vitamin B12 metabolic pathway were included for genotyping by the SNPscan technique among 454 PTB patients and 467 controls. Our results found that vitamin B12 level was significantly reduced in PTB patients when compared with controls. There was no significant association between *TCN1* rs526934, *TCN2* rs1801198, *CUBN* rs7906242, rs10904861, rs1801222, *MMACHC* rs10789465, *FUT6* rs3760776, rs3760775, *MUT* rs9473555, rs9381784 variants, and PTB susceptibility. *TCN2* rs1801198 CC genotype, C allele was significantly associated with hypoproteinemia in PTB patients. In *CUBN*, rs7906242 GG genotype, G allele, rs10904861 TT genotype, and T allele were significantly related to the decreased frequency of sputum smear-positive, and rs10904861 variant affected the occurrence of drug resistance in PTB patients. In addition, the increased frequency of *CUBN* rs1801222 AA genotype was significantly associated with leukopenia. The decreased frequency of *MUT* rs9473555 CC genotype was found in the PTB patients with hypoproteinemia. However, vitamin B12 expression was not associated with the genotype distribution of above SNPs. In conclusion, vitamin B12 level was significantly decreased in PTB patients and genetic variants in vitamin B12 metabolic genes were not contributed to PTB susceptibility. Several SNPs in *TCN2*, *CUBN*, and *MUT* gene might associate with multiple clinical manifestations in PTB.

## Introduction

Tuberculosis (TB) is a common, serious infectious disease caused by Mycobacterium tuberculosis (MTB) and remains a major threat to public health in many countries (1). There are approximately 9.9 million new incident TB patients in 2021 around the world (2). The people infected with MTB finally develop the possible outcomes as follows: MTB clearance, primary TB, latent tuberculosis infection (LTBI), and active TB (3). Many studies indicated that the risk of developing TB was strongly associated with host-pathogen interactions, external environment, and genetic factor (4). Although genetic variations in multiple genes had been found to be closely associated with TB susceptibility, it was interesting to continue to explore the role of genetic variations in TB susceptibility, which could be helpful in developing appropriate approaches to TB prevention, diagnosis, and treatment (5, 6).

Malnutrition was a risk factor for the progression of active TB and an important predictor of recurrence among TB patients (7). Because of the important role of vitamins in host nutrition and immunity, and several vitamins were essential for the survival and virulence of most organisms, including mycobacteria (8, 9). Hence, vitamin deficiency had the ability to influence the host immunity to a variety of infectious diseases. In addition, altered vitamin statuses were associated with various viral infections such as human immunodeficiency virus, influenza, and bacterial infections including dental caries and TB (10, 11). Recent meta-analysis suggested that vitamins A, D, and E expression levels were significantly lower in the TB patients than that in the control group (12).

Vitamin B12 was an essential water-soluble micronutrient to host maintain health, and vitamin B12 deficiency was linked to the development of many diseases (13). Vitamin B12 also played important roles in the development of pulmonary tuberculosis (PTB), and detecting the serum vitamin B12 and vitamin A levels could be used as an effective measure for identifying active PTB and monitoring the efficacy of PTB treatment (14). Studies had shown that the role of genetic influence on vitamin B12 expression was considerable and genetic variations might alter vitamin B12 tissue status through a variety of mechanisms (15, 16). Many genes associated with vitamin B12 metabolic pathway, such as *transcobalamin 1* (*TCN1*), *TCN2*, *fucosyltransferase 6* (*FUT6*), and *cubulin* (*CUBN*), were involved in the host susceptibility to many diseases by affecting the vitamin B12 level (17–19). However, there were no studies to analyze the relationship of these genes and PTB susceptibility. Furthermore, another report showed that the clinical manifestation and progression of PTB patients might be impacted by the host genetic variation (20). Therefore, we conducted this study to explore the effects of vitamin B12 level and vitamin B12 metabolic pathway genes (*TCN1*, *TCN2*, *CUBN*, *MMACHC*, *FUT6*, and *MUT*) variation on PTB susceptibility, clinical manifestations in a Chinese Han population.

## Materials and methods

### Study participants

The current study enrolled 80 PTB patients and 84 normal controls to detect the vitamin B12 level, and a total of 454 PTB patients and 467 normal controls were included for analyzing the association between vitamin B12 metabolic genes variation and PTB susceptibility. All PTB patients were recruited from Anhui Chest Hospital and diagnosed by clinical experts according to the following criteria: suspicious clinical symptoms, chest radiography, sputum and/or bronchoalveolar lavage fluid MTB culture, microscopy for acid fast bacilli (AFB), and effect of anti-TB treatment. The PTB patients, which were accompanied by HIV positivity, hepatitis, malignancy, and immunodeficiency, were finally excluded from this study. This study selected the healthy individuals with no history of TB, HIV positivity, malignant tumor, or other infectious diseases from the health examine center in the same area as normal controls.

The study was approved by the Medical Ethics Committee of Anhui Medical University (approval number 20200250), and the informed consent of each subject was obtained. Then, we collected peripheral blood samples of all study subjects and the clinical data and laboratory indicators of PTB patients with the support of experienced clinicians.

### Enzyme-linked immunosorbent assay

In this study, the plasma was obtained by Ficoll-Hypaque density gradient centrifugation from 2 ml of peripheral blood among 80 PTB patients and 84 normal controls. Then, we adopted Enzyme-linked immunosorbent assay (ELISA) kits (MyBioSource Inc., San Diego, CA, USA) to detect plasma vitamin B12 expression level of these study participants. The result was expressed as picomole per liter.

### Single nucleotide polymorphism selection

Six vitamin B12 metabolic genes, including *TCN1*, *TCN2*, *CUBN*, *MMACHC*, *FUT6*, and *MUT*, were selected for analyses in this study. We first identified some specific single nucleotide polymorphisms (SNPs) associated with human disease susceptibility by searching the studies regarding the association of vitamin B12 metabolic genes polymorphisms with human disease. Then, the genotype data on these six genes of Han Chinese people Beijing were obtained from Ensembl Genome Browser 85 and CHBS_1000g, and then we selected the tag SNPs with the HaploView 4.0 software (Cambridge, MA). All the selected SNPs must satisfy the following conditions: minor allele frequency (MAF) ≥ 0.05 in CHB and *r* ^2^ threshold > 0.8. Finally, we selected one SNP (rs526934) of *TCN1*, one SNP (rs1801198) of *TCN2*, three SNPs (rs7906242, rs10904861, and rs1801222) of *CUBN*, one SNP (rs10789465) of *MMACHC*, two SNPs (rs3760776 and rs3760775) of *FUT6*, and two SNPs (rs9473555 and rs9381784) of *MUT* for genotyping.

### DNA extraction and genotyping

About 5 ml of peripheral blood samples were drawn from subjects and used to extract genomic DNA by Flexi Gene-DNA Kit (Qiagen, Valencia, CA). Genotyping for above selected SNP was performed using the SNPscan Kit, with the technical support of the Center for Genetic and Genomic Analysis (Genesky Biotechnologies Inc., Shanghai, China). The SNPscan assay was a rapid multiplex genetic screening system, and its basic principle was to recognize SNP alleles by using the high specificity of ligase binding reactions (21). The specific process of SNPscan genotyping was mentioned in previous study (22). The sample could be included in the final analysis, only when all SNPs were successfully genotyped in this sample.

### Statistical analysis

Hardy–Weinberg equilibrium test was performed in normal controls using Chi-square test. Chi-square test was also used to compare the differences in genotype, allele frequencies differences of all SNPs between the PTB patients and normal controls. Odds ratios (*ORs*) and 95% confidence intervals (*CIs*) were calculated by logistic regression models. We analyzed the associations of each SNP with the risk for PTB under dominant, recessive model, and conducted haplotype analysis using SHEsis software (23). The vitamin B12 expression level was shown as *M* ± *SD*, and the differences in vitamin B12 expression level between two and three groups were analyzed by *t*-test and ANOVA, respectively. Statistical analyses were performed with SPSS 23.0, and a two-sided *P*-value of less than 0.05 was considered as the threshold of statistical significance. In SNP analysis, Bonferroni correction was adopted for multiple testing, and *P*-value of less than 0.005 (0.05/10) was considered as a significant level.

## Results

### The plasma vitamin B12 level in PTB patients and normal controls

**Figure 1:**
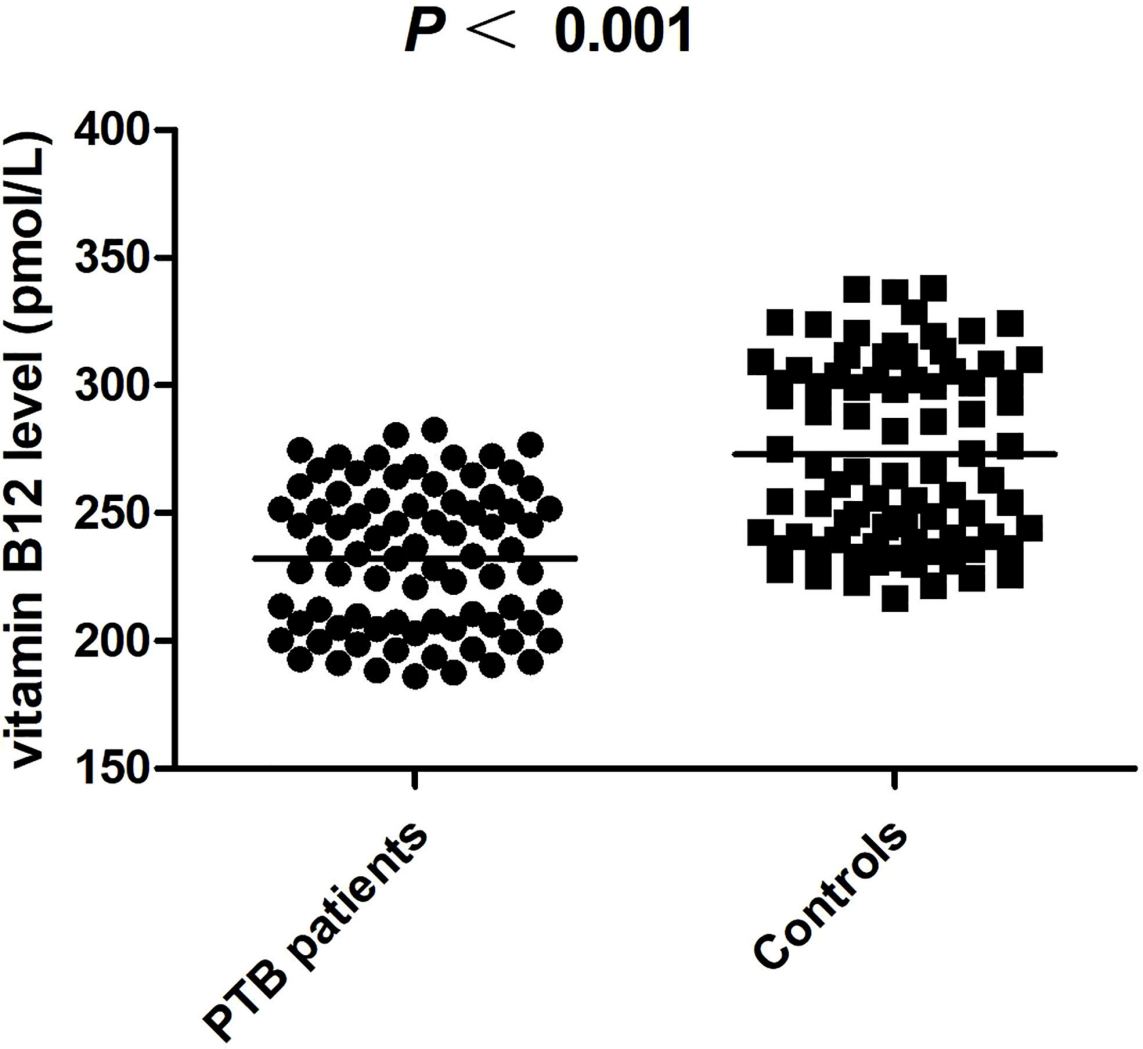
The plasma level of vitamin B12 in PTB patients and controls.

**Figure 2:**
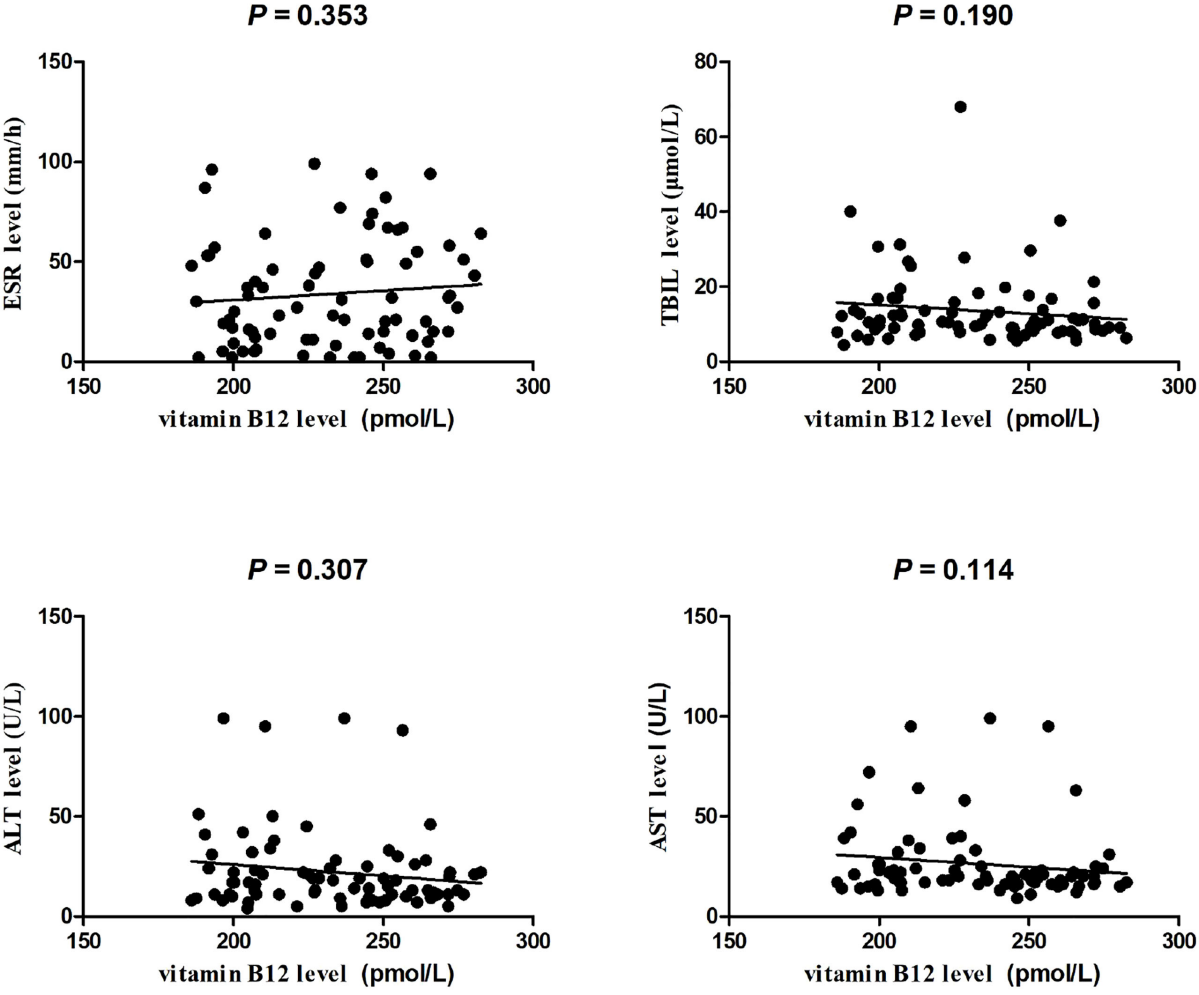
The correlation between ESR, TBIL, ALT, AST levels, and plasma vitamin B12 level in patients with PTB.

This investigated case control included 80 PTB patients (25 women and 55 men, mean age was 49.71 ± 19.08 years) and 84 normal controls (31 women and 53 men, mean age was 50.76 ± 9.69 years). As illustrated in **Figure 1**, the plasma vitamin B12 level was significantly decreased in PTB patients than normal controls (*P* <0.001). The association between vitamin B12 level and several clinical features of PTB patients was also analyzed. We found that vitamin B12 level was not associated with fever, drug resistant, DILI, pulmonary infection, and so forth in PTB patients (**Table S1**). In addition, no significant correlation was found between vitamin B12 level with ESR, TBIL, ALT, and AST in PTB (**Figure 2**).

### Association of vitamin B12 metabolic genes polymorphisms with PTB risk

In this study, the average age of 454 PTB patients, including 194 women and 260 men, was 45.35 + 17.75 years, whereas the mean age of 467 normal controls, including 264 women and 203 men, was 43.37 + 13.87 years. We found that all SNP genotype distributions in normal controls were conformed to Hardy–Weinberg equilibrium, and the results of allele and genotype frequencies of these SNPs were shown in **Table 1**.

**Table 1:** Association of vitamin B12 metabolic genes polymorphisms with PTB risk.

SNP	Analyze model		PTB patients	Controls	*P-*value			*OR* (95% *CI*)
*TCN1*								
rs526934	Genotypes	GG	24 (5.29)	27 (5.78)	0.785			0.923 (0.519,1.641)
		AG	170(37.44)	170 (36.4)	0.786			1.038 (0.791,1.364)
		AA	260 (57.27)	270(57.82)	Reference			
	Alleles	G	218 (24.01)	224 (23.98)	0.990			1.000 (0.950,1.052)
		A	690 (75.99)	710 (76.02)	Reference			
	Dominant model	AA	260 (57.27)	270 (57.82)	0.867			1.013 (0.872,1.177)
		AG+GG	194 (42.73)	197 (42.18)	Reference			
	Recessive model	GG	24 (5.29)	27 (5.78)	0.743			0.914 (0.536,1.560)
		AG+AA	430 (94.71)	440 (94.22)	Reference			
*TCN2*								
rs1801198	Genotypes	CC	90 (19.82)	92 (19.7)	0.387			0.848 (0.584,1.232)
		GC	214 (47.14)	245 (52.46)	0.067			0.757 (0.562,1.020)
		GG	150 (33.04)	130 (27.84)	Reference			
	Alleles	C	394 (43.39)	429 (45.93)	0.273			0.945 (0.853,1.064)
		G	514 (56.61)	505 (54.07)	Reference			
	Dominant model	GG	150 (33.04)	130 (27.84)	0.086			1.187 (0.975,1.444)
		GC+CC	304 (66.96)	337(72.16)	Reference			
	Recessive model	CC	90 (19.82)	92 (19.7)	0.962			0.998 (0.936,1.065)
		GC+GG	364 (80.18)	375 (80.3)	Reference			
*CUBN*								
rs7906242	Genotypes	GG	2 7 (5.95)	26 (5.57)	0.743			1.099 (0.625,1.934)
		GA	171 (37.67)	170 (36.4)	0.651			1.065 (0.811,1.498)
		AA	256 (56.39)	271 (58.03)	Reference			
	Alleles	G	225 (24.78)	222 (23.77)	0.613			1.043 (0.887,1.225)
		A	683 (75.22)	712 (76.23)	Reference			
	Dominant model	AA	256 (56.39)	271 (58.03)	0.615			0.972 (0.869,1.087)
		GA+GG	198 (43.61)	196 (41.97)	Reference			
	Recessive model	GG	27 (5.95)	26 (5.57)	0.805			1.068 (0.633,1.802)
		GA+AA	427 (94.05)	441 (94.43)	Reference			
rs10904861	Genotypes	TT	12 (2.64)	12 (2.57)	0.872			1.069 (0.474,2.415)
		CT	125 (27.53)	116 (24.84)	0.347			1.152 (0.858,1.548)
		CC	317 (69.82)	339 (72.59)	Reference			
	Alleles	T	149 (16.41)	140 (14.99)	0.402			1.095 (0.886,1.353)
		C	759 (83.59)	794 (85.01)	Reference			
	Dominant model	CC	317 (69.82)	339 (72.59)	0.354			0.962 (0.886,1.044)
		CT+TT	137 (30.18)	128 (27.41)	Reference			
	Recessive model	TT	12 (2.64)	12 (2.57)	0.944			1.029 (0.467,2.266)
		CT+CC	442 (97.36)	455 (97.43)	Reference			
rs1801222	Genotypes	AA	17 (3.74)	19 (4.07)	0.753			0.898 (0.458,1.759)
		AG	124 (27.31)	134 (28.69)	0.615			0.990 (0.695,1.241)
		GG	313 (68.94)	314 (67.24)	Reference			
	Alleles	A	158 (17.4)	172 (18.42)	0.570			1.014 (0.970,1.060)
		G	750 (82.6)	762 (81.58)	Reference			
	Dominant model	GG	313 (68.94)	314 (67.24)	0.579			1.016 (0.926,1.116)
		AG+AA	141 (31.06)	153 (32.76)	Reference			
	Recessive model	AA	17 (3.74)	19 (4.07)	0.800			0.724 (0.378,1.386)
		AG+GG	437 (96.26)	448 (95.93)	Reference			
*MMACHC*								
rs10789465	Genotypes	TT	61 (13.44)	69 (14.78)	0.345			0.822 (0.547,1.234)
		TC	222 (48.9)	239 (51.18)	0.310			0.864 (0.651,1.146)
		CC	171 (37.67)	159 (34.05)	Reference			
	Alleles	T	344 (37.89)	377 (40.36)	0.276			0.939 (0.837,1.052)
		C	564 (62.11)	557 (59.64)	Reference			
	Dominant model	CC	171 (37.67)	159 (34.05)	0.252			1.106 (0.930,1.315)
		TC+TT	283 (62.33)	308 (65.95)	Reference			
	Recessive model	TT	61 (13.44)	69 (14.78)	0.560			0.909 (0.661,1.251)
		TC+CC	393 (86.56)	398 (85.22)	Reference			
*FUT6*								
rs3760775	Genotypes	TT	35 (7.71)	26 (5.57)	0.151			1.480 (0.867,2.529)
		GT	168 (37)	165 (35.33)	0.420			1.120 (0.851,1.473)
		GG	251 (55.29)	276 (59.1)	Reference			
	Alleles	T	238 (26.21)	217 (23.23)	0.138			1.128 (0.962,1.324)
		G	670 (73.79)	717 (76.77)	Reference			
	Dominant model	GG	251 (55.29)	276 (59.1)	0.242			0.935 (0.836,1.046)
		GT+TT	203 (44.71)	191 (40.9)	Reference			
	Recessive model	TT	35 (7.71)	26 (5.57)	0.191			1.385 (0.848,2.262)
		GT+GG	419 (92.29)	441 (94.43)	Reference			
rs3760776	Genotypes	AA	7 (1.54)	6 (1.28)	0.703			1.239 (0.412,3.722)
		GA	92 (20.26)	84 (17.99)	0.369			1.163 (0.837,1.617)
		GG	355 (78.19)	377 (80.73)	Reference			
	Alleles	A	106 (11.67)	96 (10.28)	0.338			1.136 (0.875,1.474)
		G	802 (88.33)	838 (89.72)	Reference			
	Dominant model	GG	355 (78.19)	377 (80.73)	0.341			0.969 (0.907,1.034)
		GA+AA	99 (21.81)	90 (19.27)	Reference			
	Recessive model	AA	7 (1.54)	6 (1.28)	0.741			1.200 (0.406,3.544)
		GA+GG	447 (98.46)	461 (98.72)	Reference			
*MUT*								
rs9473555	Genotypes	CC	20 (4.41)	16 (3.43)	0.401			1.337 (0.679,2.633)
		GC	161(35.46)	159 (34.05)	0.569			1.083 (0.823,1.425)
		GG	273 (60.13)	292 (62.53)	Reference			
	Alleles	C	201 (22.14)	191 (20.45)	0.377			1.082 (0.908,1.290)
		G	707 (77.86)	743 (79.55)	Reference			
	Dominant model	GG	273 (60.13)	292 (62.53)	0.456			0.962 (0.868,1.066)
		GC+CC	181 (39.87)	175 (37.47)	Reference			
	Recessive model	CC	20 (4.41)	16 (3.43)	0.443			1.286 (0.675,2.450)
		GC+GG	434 (95.59)	451 (96.57)	Reference			
rs9381784	Genotypes	CC	87 (19.16)	93 (19.91)	0.513			0.882 (0.604,1.286)
		TC	228 (49.14)	243 (50.03)	0.421			0.884 (0.656,1.193)
		TT	139 (30.62)	131 (28.05)	Reference			
	Alleles	C	402 (44.27)	429 (45.93)	0.475			0.964 (0.871,1.068)
		T	506 (55.73)	505 (54.07)	Reference			
	Dominant model	TT	139 (30.62)	131 (28.05)	0.393			1.091 (0.893,1.334)
		TC+CC	315 (69.38)	336 (71.95)	Reference			
	Recessive model	CC	87 (19.16)	93 (19.91)	0.774			0.962 (0.740,1.251)
		TC+TT	367 (80.84)	374 (80.08)	Reference			

P-value was corrected by Bonferroni correction (0.05/10), P < 0.005 was considered as statistical significance.

There was no significant association between *TCN1* rs526934, *TCN2* rs1801198 variants, and PTB susceptibility (all *P* >0.005). Regarding *CUBN* gene, no significant difference in allele frequencies of rs7906242, rs10904861, and rs1801222 was observed between PTB patients and controls, and the same result was also observed in the genotype frequencies. We compared the differences in *FUT6* rs3760775, rs3760776 variants genotype, and allele frequencies among PTB patients and controls, and none of these differences were statistically significant. Similarly, there were no significant differences in allele and genotype distributions of *MMACHC* rs10789465, *MUT* rs9473555, rs9381784 polymorphism between PTB patients and controls (all *P* > 0.005). Moreover, we did not detect significant associations between these SNPs and PTB susceptibility under dominant and recessive model.

### Association of vitamin B12 metabolic genes polymorphisms with clinical features among PTB patients

We also analyzed whether vitamin B12 metabolic gene SNPs influenced the clinical manifestations, such as drug resistance, DILI, pulmonary infection, and hypoproteinemia, of PTB patients (**Table 2**). Our results found that PTB patients with *TCN2* rs1801198 CC genotype, C allele were likely to suffer from hypoproteinemia (*P* = 0.013 and *P* = 0.004, respectively), whereas the patient with *MUT* rs9473555 CC genotype was less likely to suffer from hypoproteinemia (*P* = 0.034). The GG genotype and G allele frequencies of *CUBN* rs10904861 variant were significantly associated with the increased risk of drug resistance in PTB patients (*P* = 0.033 and *P* = 0.029, respectively). Regarding the sputum smear results, the results demonstrated that *CUBN* rs7906242 GG genotype, G allele, rs10904861 TT genotype, and T allele frequencies were significantly decreased in PTB patients with sputum smear-positive when compared with PTB patients with sputum smear-negative (*P* = 0.003, *P* = 0.001, *P* = 0.019, and *P* = 0.009, respectively). In addition, the increased frequency of rs1801222 AA genotype was significantly associated with leukopenia in PTB patients (*P* < 0.001). No significant association was found between *FUT6*, *TCN1*, and *MUT* gene variations and the clinical features of PTB.

**Table 2:** Association of vitamin B12 metabolic genes polymorphisms with the clinical manifestations of PTB.

SNP	Allele	Clinical features	Group	Genotypes n (%)			*P*-value	Alleles n (%)		*P-*value
	(M/m)			MM	Mm	mm		M	m	
*TCN1*										
rs526934	A/G	fever	+	35 (50.72)	31 (44.93)	3 (4.35)	0.375	101 (73.19)	37 (26.81)	0.403
			–	225 (58.44)	139 (36.1)	21 (5.45)		589 (76.49)	181 (23.51)	
		drug resistance	+	39 (53.42)	31 (42.47)	3 (4.11)	0.595	109 (74.66)	37 (25.34)	0.680
			–	221 (58.01)	139 (36.48)	21 (5.51)		581 (76.25)	181 (23.75)	
		DILI	+	36 (54.55)	29 (43.94)	1 (1.52)	0.218	101 (76.52)	31 (23.48)	0.879
			–	224 (57.73)	141 (36.34)	23 (5.93)		589 (75.9)	187 (24.1)	
		pulmonary infection	+	45 (55.56)	30 (37.04)	6 (7.41)	0.640	120 (74.07)	42 (25.93)	0.529
			–	215 (57.64)	140 (37.53)	18 (4.83)		570 (76.41)	176 (23.59)	
		hypoproteinemia	+	19 (48.72)	19 (48.72)	1 (2.56)	0.274	57 (73.08)	21 (26.92)	0.529
			–	241 (58.07)	151 (36.39)	23 (5.54)		633 (76.27)	197 (23.73)	
		leukopenia	+	18 (58.06)	13 (41.94)	0 (0)	0.379	49 (79.03)	13 (20.97)	0.561
			–	242 (57.21)	157 (37.12)	24 (5.67)		641 (75.77)	205 (24.23)	
		sputum smear	+	75 (60)	42 (33.6)	8 (6.4)	0.791	192 (76.8)	58 (23.2)	0.812
			–	167 (57.59)	107 (36.9)	16 (5.52)		441 (76.03)	139 (23.97)	
*TCN2*										
rs1801198	G/C	fever	+	26 (37.68)	32 (46.38)	11 (15.94)	0.560	84 (60.87)	54 (39.13)	0.273
			–	124 (32.21)	182 (47.27)	79 (20.52)		430 (55.84)	340 (44.16)	
		drug resistance	+	24 (32.88)	36 (49.32)	13 (17.81)	0.875	84 (57.53)	62 (42.47)	0.805
			–	126 (33.07)	178 (46.72)	77 (20.21)		430 (56.43)	332 (43.57)	
		DILI	+	17 (25.76)	32 (48.48)	17 (25.76)	0.267	66 (50)	66 (50)	0.098
			–	133 (34.28)	182 (46.91)	73 (18.81)		448 (57.73)	328 (42.27)	
		pulmonary infection	+	27 (33.33)	39 (48.15)	15 (18.52)	0.947	93 (57.41)	69 (42.59)	0.821
			–	123 (32.98)	175 (46.92)	75 (20.11)		421 (56.43)	325 (43.57)	
		hypoproteinemia	+	5 (12.82)	22 (56.41)	12 (30.77)	**0.013**	32 (41.03)	46 (58.97)	**0.004**
			–	145 (34.94)	192 (46.27)	78 (18.8)		482 (58.07)	348 (41.93)	
		leukopenia	+	12 (38.71)	13 (41.94)	6 (19.35)	0.772	37 (59.68)	25 (40.32)	0.613
			–	138 (32.62)	201 (47.52)	84 (19.86)		477 (56.38)	369 (43.62)	
		sputum smear	+	41 (32.8)	61 (48.8)	23 (18.4)	0.716	143 (57.2)	107 (42.8)	0.654
			–	95 (32.76)	132 (45.52)	63 (21.72)		322 (55.52)	258 (44.48)	
*CUBN*										
rs7906242	A/G	fever	+	43 (62.32)	22 (31.88)	4 (5.8)	0.540	108 (78.26)	30 (21.74)	0.369
			–	213 (55.32)	149 (38.7)	23 (5.97)		575 (74.68)	195 (25.32)	
		drug resistance	+	46 (63.01)	22 (30.14)	5 (6.85)	0.349	114 (78.08)	32 (21.92)	0.382
			–	210 (55.12)	149 (39.11)	22 (5.77)		569 (74.67)	193 (25.33)	
		DILI	+	34 (51.52)	28 (42.42)	4 (6.06)	0.673	96 (72.73)	36 (27.27)	0.473
			–	222 (57.22)	143 (36.86)	23 (5.93)		587 (75.64)	189 (24.36)	
		pulmonary infection	+	48 (59.26)	31 (38.27)	2 (2.47)	0.340	127 (78.4)	35 (21.6)	0.302
			–	208 (55.76)	140 (37.53)	25 (6.7)		556 (74.53)	190 (25.47)	
		hypoproteinemia	+	21 (53.85)	16 (41.03)	2 (5.13)	0.894	58 (74.36)	20 (25.64)	0.854
			–	235 (56.63)	155 (37.35)	25 (6.02)		625 (75.3)	205 (24.7)	
		leukopenia	+	19 (61.29)	11 (35.48)	1 (3.23)	0.741	49 (79.03)	13 (20.97)	0.471
			–	237 (56.03)	160 (37.83)	26 (6.15)		634 (74.94)	212 (25.06)	
		sputum smear	+	81 (64.8)	43 (34.4)	1 (0.8)	**0.003**	205 (82.00)	45 (18.00)	**0.001**
			–	149 (51.38)	117 (40.34)	24 (8.28)		415 (71.55)	165 (28.45)	
rs10904861	C/T	fever	+	50 (72.46)	15 (21.74)	4 (5.8)	0.126	115 (83.33)	23 (16.67)	0.929
			–	267 (69.35)	110 (28.57)	8 (2.08)		644 (83.64)	126 (16.36)	
		drug resistance	+	60 (82.19)	11 (15.07)	2 (2.74)	**0.033**	131 (89.73)	15 (10.27)	**0.029**
			–	257 (67.45)	114 (29.92)	10 (2.62)		628 (82.41)	134 (17.59)	
		DILI	+	44 (66.67)	19 (28.79)	3 (4.55)	0.546	107 (81.06)	25 (18.94)	0.396
			–	273 (70.36)	106 (27.32)	9 (2.32)		652 (84.02)	124 (15.98)	
		pulmonary infection	+	58 (71.6)	23 (28.4)	0 (0)	0.262	139 (85.8)	23 (14.2)	0.402
			–	259 (69.44)	102 (27.35)	12 (3.22)		620 (83.11)	126 (16.89)	
		hypoproteinemia	+	25 (64.1)	13 (33.33)	1 (2.56)	0.697	63 (80.77)	15 (19.23)	0.482
			–	292 (70.36)	112 (26.99)	11 (2.65)		696 (83.86)	134 (16.14)	
		leukopenia	+	23 (74.19)	8 (25.81)	0 (0)	0.605	54 (87.1)	8 (12.9)	0.440
			–	294 (69.5)	117 (27.66)	12 (2.84)		705 (83.33)	141 (16.67)	
		sputum smear-positive	+	96 (76.8)	29 (23.2)	0 (0)	**0.019**	221 (88.4)	29 (11.6)	**0.009**
			–	192 (66.21)	86 (29.66)	12 (4.14)		470 (81.03)	110 (18.97)	
rs1801222	G/A	fever	+	52 (75.36)	13 (18.84)	4 (5.8)	0.170	117 (84.78)	21 (15.22)	0.463
			–	261 (67.79)	111 (28.83)	13 (3.38)		633 (82.21)	137 (17.79)	
		drug resistance	+	47 (64.38)	25 (34.25)	1 (1.37)	0.212	119 (81.51)	27 (18.49)	0.704
			–	266 (69.82)	99 (25.98)	16 (4.2)		631 (82.81)	131 (17.19)	
		DILI	+	50 (75.76)	15 (22.73)	1 (1.52)	0.343	115 (87.12)	17 (12.88)	0.138
			–	263 (67.78)	109 (28.09)	16 (4.12)		635 (81.83)	141 (18.17)	
		pulmonary infection	+	50 (61.73)	27 (33.33)	4 (4.94)	0.297	127 (78.4)	35 (21.6)	0.119
			–	263 (70.51)	97 (26.01)	13 (3.49)		623 (83.51)	123 (16.49)	
		hypoproteinemia	+	31 (79.49)	7 (17.95)	1 (2.56)	0.330	69 (88.46)	9 (11.54)	0.153
			–	282 (67.95)	117 (28.19)	16 (3.86)		681 (82.05)	149 (17.95)	
		leukopenia	+	22 (70.97)	3 (9.68)	6 (19.35)	**0.000**	47 (75.81)	15 (24.19)	0.144
			–	291 (68.79)	121 (28.61)	11 (2.6)		703 (83.1)	143 (16.9)	
		sputum smear-positive	+	81 (64.8)	39 (31.2)	5 (4)	0.665	201 (80.4)	49 (19.6)	0.382
			–	201 (69.31)	79 (27.24)	10 (3.45)		481 (82.93)	99 (17.07)	
*MMACHC*										
rs10789465	C/T	fever	+	24 (34.78)	39 (56.52)	6 (8.7)	0.285	87 (63.04)	51 (36.96)	0.807
			–	147 (38.18)	183 (47.53)	55 (14.29)		477 (61.95)	293 (38.05)	
		drug resistance	+	22 (30.14)	41 (56.16)	10 (13.7)	0.324	85 (58.22)	61 (41.78)	0.290
			–	149 (39.11)	181 (47.51)	51 (13.39)		479 (62.86)	283 (37.14)	
		DILI	+	20 (30.3)	40 (60.61)	6 (9.09)	0.113	80 (60.61)	52 (39.39)	0.699
			–	151 (38.92)	182 (46.91)	55 (14.18)		484 (62.37)	292 (37.63)	
		pulmonary infection	+	33 (40.74)	34 (41.98)	14 (17.28)	0.317	100 (61.73)	62 (38.27)	0.911
			–	138 (37)	188 (50.4)	47 (12.6)		464 (62.2)	282 (37.8)	
		hypoproteinemia	+	18 (46.15)	17 (43.59)	4 (10.26)	0.501	53 (67.95)	25 (32.05)	0.267
			–	153 (36.87)	205 (49.4)	57 (13.73)		511 (61.57)	319 (38.43)	
		leukopenia	+	10 (32.26)	18 (58.06)	3 (9.68)	0.554	38 (61.29)	24 (38.71)	0.890
			–	161 (38.06)	204 (48.23)	58 (13.71)		526 (62.17)	320 (37.83)	
		sputum smear-positive	+	45 (36)	60 (48)	20 (16)	0.770	150 (60)	100 (40)	0.512
			–	111 (38.28)	140 (48.28)	39 (13.45)		362 (62.41)	218 (37.59)	
*FUT6*										
rs3760775	G/T	fever	+	38 (55.07)	28 (40.58)	3 (4.35)	0.478	104 (75.36)	34 (24.64)	0.648
			–	213 (55.32)	140 (36.36)	32 (8.31)		566 (73.51)	204 (26.49)	
		drug resistance	+	46 (63.01)	20 (27.4)	7 (9.59)	0.173	112 (76.71)	34 (23.29)	0.381
			–	205 (53.81)	148 (38.85)	28 (7.35)		558 (73.23)	204 (26.77)	
		DILI	+	39 (59.09)	21 (31.82)	6 (9.09)	0.620	99 (75)	33 (25)	0.732
			–	212 (54.64)	147 (37.89)	29 (7.47)		571 (73.58)	205 (26.42)	
		pulmonary infection	+	40 (49.38)	36 (44.44)	5 (6.17)	0.301	116 (71.6)	46 (28.4)	0.486
			–	211 (56.57)	132 (35.39)	30 (8.04)		554 (74.26)	192 (25.74)	
		hypoproteinemia	+	16 (41.03)	21 (53.85)	2 (5.13)	0.074	53 (67.95)	25 (32.05)	0.220
			–	235 (56.63)	147 (35.42)	33 (7.95)		617 (74.34)	213 (25.66)	
		leukopenia	+	17 (54.84)	13 (41.94)	1 (3.23)	0.581	47 (75.81)	15 (24.19)	0.708
			–	234 (55.32)	155 (36.64)	34 (8.04)		623 (73.64)	223 (26.36)	
		sputum smear-positive	+	67 (53.6)	50 (40)	8 (6.4)	0.512	184 (73.6)	66 (26.4)	0.964
			–	162 (55.86)	102 (35.17)	26 (8.97)		426 (73.45)	154 (26.55)	
rs3760776	G/A	fever	+	55 (79.71)	14 (20.29)	0 (0)	0.528	124 (89.86)	14 (10.14)	0.544
			–	300 (77.92)	78 (20.26)	7 (1.82)		678 (88.05)	92 (11.95)	
		drug resistance	+	57 (78.08)	15 (20.55)	1 (1.37)	0.990	129 (88.36)	17 (11.64)	0.990
			–	298 (78.22)	77 (20.21)	6 (1.57)		673 (88.32)	89 (11.68)	
		DILI	+	52 (78.79)	14 (21.21)	0 (0)	0.541	118 (89.39)	14 (10.61)	0.679
			–	303 (78.09)	78 (20.1)	7 (1.8)		684 (88.14)	92 (11.86)	
		pulmonary infection	+	64 (79.01)	17 (20.99)	0 (0)	0.460	145 (89.51)	17 (10.49)	0.606
			–	291 (78.02)	75 (20.11)	7 (1.88)		657 (88.07)	89 (11.93)	
		hypoproteinemia	+	32 (82.05)	6 (15.38)	1 (2.56)	0.647	70 (89.74)	8 (10.26)	0.683
			–	323 (77.83)	86 (20.72)	6 (1.45)		732 (88.19)	98 (11.81)	
		leukopenia	+	24 (77.42)	7 (22.58)	0 (0)	0.739	55 (88.71)	7 (11.29)	0.922
			–	331 (78.25)	85 (20.09)	7 (1.65)		747 (88.3)	99 (11.7)	
		sputum smear-positive	+	100 (80)	24 (19.2)	1 (0.8)	0.602	224 (89.6)	26 (10.4)	0.409
			–	224 (77.24)	60 (20.69)	6 (2.07)		508 (87.59)	72 (12.41)	
*MUT*										
rs9473555	G/C	fever	+	21 (30.43)	34 (49.28)	14 (20.29)	0.966	76 (55.07)	62 (44.93)	0.867
			–	118 (30.65)	194 (50.39)	73 (18.96)		430 (55.84)	340 (44.16)	
		drug resistance	+	15 (20.55)	42 (57.53)	16 (21.92)	0.125	72 (49.32)	74 (50.68)	0.089
			–	124 (32.55)	186 (48.82)	71 (18.64)		434 (56.96)	328 (43.04)	
		DILI	+	23 (34.85)	30 (45.45)	13 (19.7)	0.666	76 (57.58)	56 (42.42)	0.644
			–	116 (29.9)	198 (51.03)	74 (19.07)		430 (55.41)	346 (44.59)	
		pulmonary infection	+	20 (24.69)	43 (53.09)	18 (22.22)	0.412	83 (51.23)	79 (48.77)	0.204
			–	119 (31.9)	185 (49.6)	69 (18.5)		423 (56.7)	323 (43.3)	
		hypoproteinemia	+	11 (28.21)	26 (66.67)	2 (5.13)	**0.034**	48 (61.54)	30 (38.46)	0.280
			–	128 (30.84)	202 (48.67)	85 (20.48)		458 (55.18)	372 (44.82)	
		leukopenia	+	11 (35.48)	13 (41.94)	7 (22.58)	0.633	35 (56.45)	27 (43.55)	0.905
			–	128 (30.26)	215 (50.83)	80 (18.91)		471 (55.67)	375 (44.33)	
		sputum smear-positive	+	36 (28.8)	67 (53.6)	22 (17.6)	0.727	139 (55.6)	111 (44.4)	0.661
			–	94 (32.41)	144 (49.66)	52 (17.93)		332 (57.24)	248 (42.76)	
rs9381784	T/C	fever	+	41 (59.42)	24 (34.78)	4 (5.8)	0.829	106 (76.81)	32 (23.19)	0.747
			–	232 (60.26)	137 (35.58)	16 (4.16)		601 (78.05)	169 (21.95)	
		drug resistance	+	42 (57.53)	27 (36.99)	4 (5.48)	0.826	111 (76.03)	35 (23.97)	0.560
			–	231 (60.63)	134 (35.17)	16 (4.2)		596 (78.22)	166 (21.78)	
		DILI	+	41 (62.12)	20 (30.3)	5 (7.58)	0.302	102 (77.27)	30 (22.73)	0.860
			–	232 (59.79)	141 (36.34)	15 (3.87)		605 (77.96)	171 (22.04)	
		pulmonary infection	+	49 (60.49)	28 (34.57)	4 (4.94)	0.957	126 (77.78)	36 (22.22)	0.977
			–	224 (60.05)	133 (35.66)	16 (4.29)		581 (77.88)	165 (22.12)	
		hypoproteinemia	+	25 (64.1)	14 (35.9)	0 (0)	0.369	64 (82.05)	14 (17.95)	0.351
			–	248 (59.76)	147 (35.42)	20 (4.82)		643 (77.47)	187 (22.53)	
		leukopenia	+	18 (58.06)	9 (29.03)	4 (12.9)	0.053	45 (72.58)	17 (27.42)	0.299
			–	255 (60.28)	152 (35.93)	16 (3.78)		662 (78.25)	184 (21.75)	
		sputum smear-positive	+	75 (60)	47 (37.6)	3 (2.4)	0.586	197 (78.8)	53 (21.2)	0.998
			–	179 (61.72)	99 (34.14)	12 (4.14)		457 (78.79)	123 (21.21)	

Bold value means P < 0.05.

### Haplotype analysis

The main haplotypes with frequencies ≥ 3% in both PTB patients and controls of *CUBN*, *FUT6*, and *MUT* genes was detected using SHEsis software in this study. The frequency distributions of these haplotypes, including five haplotypes (ACG, ATG, GCA, and GCG) for *CUBN*, four main haplotypes (GA, GG, TA, and TG) for *FUT6*, three main haplotypes (CC, CG, and TG) for *MUT*, were summarized in **Table 3**. None of these haplotypes was significantly associated with the risk of PTB by comparing the differences in haplotype frequency between PTB patients and normal controls.

**Table 3:** Haplotype analysis of *CUBN*, *FUT6*, and *MUT* genes in PTB patients and controls.

Haplotype	PTB [n(%)]	Controls [n(%)]	*P*-value	*OR* (95% CI)
*CUBN* rs7906242-rs10904861-rs1801222				
ACG	65.88 (7.3)	68.54 (7.3)	0.932	0.985 (0.693,1.400)
ATG	122.80 (13.5)	113.42 (12.1)	0.387	1.129 (0.858,1.485)
GCA	120.26 (13.2)	131.96 (14.1)	0.563	0.924 (0.708,1.207)
GCG	557.22 (61.4)	574.54 (61.5)	0.883	0.986 (0.810,1.198)
*FUT6* rs3760775-rs3760776				
GA	29.03 (3.2)	29.76 (3.2)	0.990	1.003 (0.597,1.687)
GG	640.97 (70.6)	676.24 (73.6)	0.153	0.862 (0.703,1.057)
TA	76.97 (8.5)	66.24 (7.1)	0.267	1.213 (0.862,1.708)
TG	161.03 (17.7)	150.76 (16.1)	0.362	1.120 (0.878,1.429)
*MUT* rs9381784- rs9473555				
CC	201.00 (22.1)	189.61 (20.3)	0.344	1.114 (0.891,1.393)
CG	201.00 (22.1)	239.39 (25.6)	0.076	0.823 (0.664,1.021)
TG	506.00 (55.7)	503.61 (53.9)	0.457	1.072 (0.892,1.288)

Frequency < 0.03 in both controls and PTB patients has been dropped.

### Associations between vitamin B12 metabolic genes polymorphisms with vitamin B12 level in PTB patients

We finally assessed the association between vitamin B12 metabolic genes (*TCN1*, *TCN2*, *CUBN*, *MMACHC*, *FUT6*, and *MUT*) variation and vitamin B12 level among 68 PTB patients. Our results showed that there was no significant difference in vitamin B12 level among their different genotypes (all *P* > 0.05) (**Table S2**).

## Discussion

Studies had suggested that vitamins might play important roles in the prevention of PTB, and genetic variation and abnormal expression of most vitamins were closely related to the susceptibility to PTB (24, 25). At present, there were many studies regarding the role of vitamins A, D, and E in the development of PTB, whereas few studies on vitamin B12. Our previous study found that some SNPs in vitamin D metabolic pathway genes were associated with susceptibility to PTB and might contribute to several clinical phenotypes of PTB patients (26). It is worth noting that MTB had the ability to regulate core metabolic functions according to B12 availability, that is, whether vitamin B12 was acquired by endogenous synthesis or through uptake from the host environment (9). Therefore, vitamin B12 played a pivotal role in the pathogenesis of PTB, whereas the mechanism still needed to be further explored. This study was the first to explore the role of vitamin B12 metabolic genes variation in the development of PTB. We finally selected 10 SNPs to examine the association between vitamin B12 metabolic genes (*TCN1*, *TCN2*, *CUBN*, *MMACHC*, *FUT6*, and *MUT*) and PTB susceptibility.

These genes were key genes in vitamin B12 metabolism, including four co-factors or regulators of vitamin B12 transport (*FUT6*, *MMACHC*, *TCN1*, and *TCN2*), one membrane transporters (CUBN), and one mitochondrial protein (MUT) (13). *TCN1* gene was located on chromosome 11 and coded the vitamin B12 binding protein (27), which facilitated the entry of vitamin B12 into the cells through receptor mediated endocytosis. The *TCN2* gene was located on chromosome 22 and had the capacity to make a vitamin B12 binding protein called transcobalamin II (TC) (28). *FUT6* gene was located on chromosome 19, which encoded a Golgi stack membrane protein, and associated with vitamin B12 deficiency (29). The *MMACHC* gene was located on chromosome region 1p34.1, and encoded a chaperone protein MMACHC (cblC protein), which could bind to vitamin B12 in the cytoplasm (30). *CUBN* gene was located on chromosome 10 and expressed in intestinal and renal epithelial cells, which was involved in the uptake of the intrinsic factor-vitamin B12 complex (31). The *MUT* gene was located on chromosome 6, and provided instructions for the formation of methylmalonyl CoA mutase, which was a mitochondrial enzyme (13). Several SNPs in these genes had been shown to mediate disease progression by affecting vitamin B12 levels.

The high level of methylmalonic acid (MMA) has been proved to be a biomarker reflecting vitamin B12 deficiency with high sensitivity and specificity; therefore, Oh *et al.* detected the expression level of MMA in TB patients (24). They found that MMA level was significantly higher in PTB patients than that in controls, suggesting that vitamin B12 deficiency existed in PTB patients. Similarly, we found that the plasma vitamin B12 level was significantly decreased in PTB patients than normal controls. We believed that vitamin B12 was closely related to the pathogenesis of PTB, and vitamin B12 metabolic gene variation might affect susceptibility to PTB by affecting vitamin B12 expression level. The rs526934 variant in *TCN1* gene was found to be associated with lower circulating vitamin B12 concentrations and an increased risk of developing gastric cancer (18). A significant association between *TCN2* gene rs1801198 and serum vitamin B12 levels was observed in a male Irish population whereas not in Caucasian populations. Moreover, *TCN2* rs1801198 and rs9606756 variations were significantly correlated with ulcerative colitis (32). Other studies also confirmed that *CUBN* gene rs1801222, *MMACHC* gene rs10789465, *FUT6* gene rs3760776, rs3760775, *MUT* gene rs9473555, and rs9381784 polymorphisms were associated with vitamin B12 concentrations (13, 29, 33). In the present study, we assessed the potential association between *TCN1* gene rs526934, *TCN2* gene rs1801198, *CUBN* gene rs7906242, rs10904861, rs1801222, *MMACHC* gene rs10789465, *FUT6* gene rs3760776, rs3760775, *MUT* gene rs9473555, rs9381784 polymorphisms, and PTB susceptibility and found that these SNPs were not contributed to PTB development. Association studies based on haplotypes of multiple markers could increase the efficiency of mapping and characterizing disease-causing genes (34); however, we still found no association between multiple haplotypes of *CUBN*, *FUT6*, *MUT* genes, and the risk of PTB. In addition, no statistically significant association between these SNPs and vitamin B12 expression level was observed in patients with PTB. The differences between our results and other studies might be due to the different race and disease characteristics. The association between vitamin B12 metabolic genes variation and vitamin B12 expression level were influenced by race, as well as disease characteristics. At present, studies on gene variation of vitamin B12 metabolic pathway and susceptibility to PTB were very limited, and different experimental methods and sample sizes might also affect the accuracy of the results. Therefore, reproducible studies were still necessary to investigate the precise role of vitamin B12-related genes variation in PTB.

In the process of PTB treatment, patients usually accompanied by pulmonary infection, hypoproteinemia, adverse drug reactions, and other adverse clinical manifestations, which were a major challenge to PTB treatment. According to one previous study, side effects had become the leading cause of unsuccessful response to treatment in PTB patients (35). Hence, it was of great significance to assess the roles of SNPs in the development of multiple clinical manifestations. Our previous studies showed that *CYP27A1* gene rs17470271 T; rs933994 T allele frequencies were respectively significantly related to leukopenia, drug resistance, and lncRNA *NEAT1* gene rs3825071 TT genotype; T allele frequencies were significantly increased in sputum smear-positive PTB patients (24, 36). In the present study, we revealed that *TCN2* gene rs1801198, *MUT* gene rs9473555 variants was significantly associated with hypoproteinemia, and *CUBN* gene rs10904861 variant was significantly corrected with the occurrence of drug resistance in PTB patients. In addition, a significant association between *CUBN* gene rs7906242 GG genotype, G allele, rs10904861 TT genotype, T allele frequencies, and sputum smear-positive was found, and *CUBN* gene rs1801222 AA genotype could affect the susceptibility of the patients to leukopenia. These findings could further improve our understanding of the roles of vitamin B12 metabolism genes in the development of PTB and contribute to making more appropriate treatment options.

In conclusion, our study demonstrated that vitamin B12 level was significantly decreased in PTB patients, and vitamin B12 metabolic pathway genes (*TCN1*, *TCN2*, *CUBN*, *MMACHC*, *FUT6*, and *MUT*) variation might not contribute to PTB susceptibility. In addition, several SNPs in *TCN2*, *CUBN*, and *MUT* genes appeared to be more prone to the occurrence of multiple clinical manifestations, including drug resistance, hypoproteinemia, and leukopenia in PTB patients. Some limitations existed in this study should be noted. First, the sample size was relatively insufficient, which might affect the accuracy of our results. Second, the mechanism of vitamin B12 metabolic genes variation in PTB susceptibility needed to be further explored. Therefore, functional and reproducible studies with larger sample sizes were required to further understand the genetic mechanisms of vitamin B12 metabolic genes in PTB development.

## Data availability statement

The original contributions presented in the study are included in the article/**Supplementary Material**. Further inquiries can be directed to the corresponding authors.

## Ethics statement

This study was reviewed and approved by the Ethics Committee of Anhui Medical University (20200250). The patients/participants provided their written informed consent to participate in this study.

## Author contributions

H-ML, T-PZ and FT designed the study. H-ML conducted the experiment. RL performed the statistical analyses. L-JW and FT participated in sample collection. T-PZ drafted the manuscript. H-ML contributed to the manuscript revision. All authors contributed to the article and approved the submitted version.

## Funding

This work was supported by grants from the National Natural Science Foundation of China (82003515), and Anhui Provincial Medical and Health Key Specialty Construction Project (No.[2021]273).

## Conflict of interest

The authors declare that the research was conducted in the absence of any commercial or financial relationships that could be construed as a potential conflict of interest.

## Publisher’s note

All claims expressed in this article are solely those of the authors and do not necessarily represent those of their affiliated organizations, or those of the publisher, the editors and the reviewers. Any product that may be evaluated in this article, or claim that may be made by its manufacturer, is not guaranteed or endorsed by the publisher.
