# Supplementary material for: Clinical relevance of vitamin B12 level and vitamin B12 metabolic gene variation in pulmonary tuberculosis
Source: Front Immunol. 2022 Oct 6;13:947897. doi: 10.3389/fimmu.2022.947897 (PMC9583150; doi:10.3389/fimmu.2022.947897)
Supplement: Supplementary file 1 [file Table_1.doc]

**Table S1** The association between vitamin B12 level and clinical features of PTB patients

| Group | +/- | N | vitamin B12 level | *P* value |
| --- | --- | --- | --- | --- |
| fever | + | 16 | 242.32±29.40 | 0.100 |
|  | - | 64 | 229.53±27.04 |  |
| drug resistant | + | 3 | 233.79±35.87 | 0.915 |
|  | - | 77 | 232.02±27.75 |  |
| DILI | + | 9 | 228.00±25.94 | 0.643 |
|  | - | 71 | 232.61±28.17 |  |
| pulmonary infection | + | 69 | 233.06±27.66 | 0.436 |
|  | - | 11 | 225.97±29.32 |  |
| hypoproteinemia | + | 62 | 232.19±28.82 | 0.952 |
|  | - | 18 | 231.74±24.79 |  |
| leukopenia | + | 7 | 234.51±34.56 | 0.811 |
|  | - | 73 | 231.86±27.37 |  |
| Sputum smear | + | 53 | 231.95±30.14 | 0.950 |
|  | - | 27 | 232.37±23.09 |  |

+/-: with/without; median (interquartile range); ^a^part of the study subjects of data missing.
