# Supplementary material for: Clinical relevance of vitamin B12 level and vitamin B12 metabolic gene variation in pulmonary tuberculosis
Source: Front Immunol. 2022 Oct 6;13:947897. doi: 10.3389/fimmu.2022.947897 (PMC9583150; doi:10.3389/fimmu.2022.947897)
Supplement: Supplementary file 2 [file Table_2.doc]

**Table S2** Association between vitamin B12 metabolic genes polymorphisms with

vitamin B12 level in PTB patients

| *TCN1* SNP | Genotypes | number | vitamin B12 level | *P* value |
| --- | --- | --- | --- | --- |
| rs526934 | GG | 1 | 271.73 | 0.340 |
|  | GA | 30 | 230.74±28.67 |  |
|  | AA | 37 | 229.68±27.48 |  |
| *TCN2* SNP | Genotypes | number | vitamin B12 level | *P* value |
| rs1801198 | CC | 16 | 231.41±25.78 | 0.937 |
|  | GC | 28 | 229.30±28.30 |  |
|  | GG | 24 | 232.03±30.25 |  |
| *CUBN* SNP | Genotypes | number | vitamin B12 level | *P* value |
| rs7906242 | AA | 4 | 224.06±22.62 | 0.738 |
|  | GA | 30 | 228.89±28.70 |  |
|  | GG | 34 | 233.22±28.51 |  |
| rs10904861 | TT | 1 | 227.07 | 0.890 |
|  | CT | 25 | 232.92±28.43 |  |
|  | CC | 42 | 229.57±28.44 |  |
| rs1801222 | AA | 3 | 222.40±43.69 | 0.865 |
|  | AG | 23 | 230.50±29.38 |  |
|  | GG | 42 | 231.51±26.91 |  |
| *MMACHC* SNP | Genotypes | number | vitamin B12 level | *P* value |
| rs10789465 | TT | 13 | 220.09±29.45 | 0.193 |
|  | TC | 30 | 236.71±27.72 |  |
|  | CC | 25 | 229.17±26.96 |  |
| *FUT6* SNP | Genotypes | number | vitamin B12 level | *P* value |
| rs3760775 | TT | 4 | 247.86±33.10 | 0.422 |
|  | GT | 27 | 231.42±29.57 |  |
|  | GG | 37 | 228.44±26.54 |  |
| rs3760776 | AA | 2 | 234.26±53.49 | 0.954 |
|  | GA | 11 | 232.63±34.24 |  |
|  | GG | 55 | 230.27±26.56 |  |
| MUT SNP | Genotypes | number | vitamin B12 level | *P* value |
| rs9473555 | CC | 1 | 207.18 | 0.615 |
|  | GC | 25 | 233.73±31.01 |  |
|  | GG | 42 | 229.22±25.84 |  |
| rs9381784 | TT | 24 | 232.82±28.20 | 0.596 |
|  | TC | 35 | 231.62±27.57 |  |
|  | CC | 9 | 221.91±31.18 |  |

Median (interquartile range)
